# Event-Based Sensing and Control for Remote Robot Guidance: An Experimental Case

**DOI:** 10.3390/s17092034

**Published:** 2017-09-06

**Authors:** Carlos Santos, Miguel Martínez-Rey, Felipe Espinosa, Alfredo Gardel, Enrique Santiso

**Affiliations:** Electronics Department, University of Alcalá, Engineering School, Campus Universitario, 28871 Alcalá de Henares, Spain; carlos.santos@uah.es (C.S.); miguel.martinez@depeca.uah.es (M.M.-R.); alfredo.gardel@uah.es (A.G.); enrique.santiso@uah.es (E.S.)

**Keywords:** event-based state estimation, covariance-based triggering, event-based Lyapunov control, practical stability, robotic remote guidance, nonlinear trajectory tracking

## Abstract

This paper describes the theoretical and practical foundations for remote control of a mobile robot for nonlinear trajectory tracking using an external localisation sensor. It constitutes a classical networked control system, whereby event-based techniques for both control and state estimation contribute to efficient use of communications and reduce sensor activity. Measurement requests are dictated by an event-based state estimator by setting an upper bound to the estimation error covariance matrix. The rest of the time, state prediction is carried out with the Unscented transformation. This prediction method makes it possible to select the appropriate instants at which to perform actuations on the robot so that guidance performance does not degrade below a certain threshold. Ultimately, we obtained a combined event-based control and estimation solution that drastically reduces communication accesses. The magnitude of this reduction is set according to the tracking error margin of a P3-DX robot following a nonlinear trajectory, remotely controlled with a mini PC and whose pose is detected by a camera sensor.

## 1. Introduction

The vast array of networked control system (NCS) and wireless sensor network (WSN) applications range from natural monitoring to ambient awareness, and from academia to industrial plants and domestic home environments [1,2,3,4]. Consequently, this field has attracted substantial research attention.

Wireless remote control of multiple robotic units in an environment with multiple sensorial modules providing partial or total information about the state of the plant is a clear example of NCS and WSN. In these contexts, wireless networks are part of the solution and the problem alike [5]. The drawbacks sharing a common link: time-varying delays [6,7], packet dropouts [8,9], packet disorder [8,10], and network bandwidth constraints [11,12].

In order to leverage the advantages of flexibility and maintenance of wireless networks and reduce their negative effects on controlled plant stability, event-based control and event-based estimation solutions have been proposed [13], most of which have been theoretically addressed or independently implemented.

### 1.1. Review of Event-Based Control in Robotics

Several studies have been published on event-based remote control of robotic units. In [14], in which a controller communicates via wireless network (IEEE 802.11 g) with a mobile robot tracking a trajectory, a nonlinear event-triggered feedback law was designed and implemented on a Khepera III robot (EPFL, Lausanne, Switzerland); and a Lyapunov function was designed based on the Cartesian reference system. However, aperiodic communication only concerned transmissions from the controller to the robot, while odometry information from robot to controller was allowed periodically. In [15], an experimental platform was developed to enable communication between a set of LEGO (Billund, Denmark) robots through a wireless network. The mobile robots obtained their position through a camera connected to a PC, which communicated with each robot using the ZigBee standard. A distributed event-triggered control was implemented to bring the robots into the desired formation. However, the PC sent the absolute position and orientation periodically to the robots. In [16], a potential field approach is combined with event-driven control to reduce the computational cost yielding advantages in terms of goal convergence and collision avoidance for robotics applications, but only simulation results are reported. In [17], an event-triggered torque control for an omnidirectional mobile robot is real-time implemented. A Lyapunov function is applied to achieve asymptotic stability, and the only feedback is odometric information from the robot. In [18], an event-based communication framework for remote operation of a robot via a bandwidth-limited network is described. The robot sends state estimation data to the operator, and the operator transmits updated control commands to the robot. Simulation results of a robotic arm highlight its potential for efficient use of limited communication resources. However, the use of an external sensor module was not considered. In [19], an approach is proposed for a wireless control system for mobile robots. The event generator block at the robot calculates the difference between the sensor signals and the reference ones. When this difference crosses a certain threshold, an event is generated, and the error is sent to the remote controller over the radio interface. In [20], a quad-rotor is controlled through an event-triggered sampling strategy. The roll and pitch angles are auto-stabilized on-board. The on-board sensor implements a mixed triggering mechanism to send via Wifi the output plant to the ground control station (remote centre), which is only responsible for the yaw angle. The results of simulation with Truetime (Lund University, Sweden) and experimentation are reported. These authors have also worked on adaptive self-triggered remote control solutions [21], as in the case of a P3-DX robot application (Omron Adept, Amherst, NH, USA), where the only sensory information is provided by an on-board odometric system.

### 1.2. Review of Event-Based Estimation and Sensing in Robotics

Other works are mainly focused on event-based estimation of robotic platforms. In [22], a balancing cube that can autonomously balance on any of its edges or corners serves as a platform for testing a distributed estimation algorithm. Experiments demonstrated that the distributed estimation algorithm combined with an event-based communication protocol yielded a significant reduction in the average number of communicated sensor measurements while keeping the cube balanced. In [23], an event-based state estimation scenario is considered where multiple distributed sensors sporadically transmit observations of a linear process to a time-varying Kalman filter via a common bus. The triggering decision is based on the estimation variance combined with a Kalman Filter. However, only the simulation results obtained with Matlab (version 2012a, MathWorks Ltd., Natick, MA, USA) are presented. In [24], an event-based state estimator (EBSE) is described consisting of an unscented Kalman filter that uses a triggering mechanism based on the estimation error covariance matrix to request measurements from the external sensors. The EBSE generates the events of the estimator module on-board the vehicle, and thus allows the sensors to remain in stand-by mode until an event is generated. A simulated example is reported where an autonomous vehicle must approach and follow a reference trajectory, but not event-scheduled control approach is considered. In [25], a state estimation problem is considered for a hidden Markov model subject to event-based sensor measurement updates sent through a reliable communication channel. An analytical expression is obtained for the probability distributions of the states conditional on all the past hybrid point- and set-valued measurement information caused by the event-triggering scheme. This was a theoretical study that did not include experimental or simulated applications.

### 1.3. Objectives

**Figure 1:**
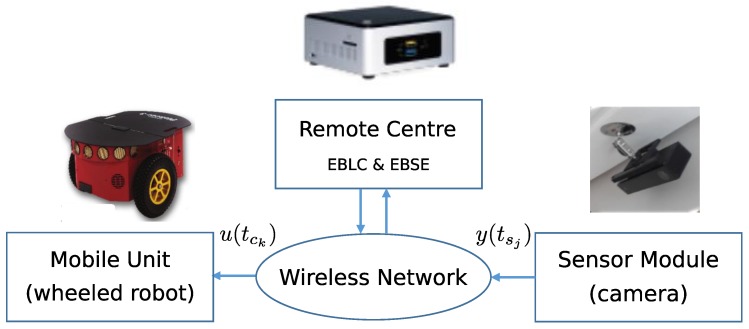
Main elements involved in event-based sensing and control for P3-DX guidance implemented on a remote PC.

The goal of our work is to integrate aperiodic sensing (EBSE) and control (event-based Lyapunov control—EBLC-) techniques to remotely control a non-holonomic robotic unit tracking nonlinear trajectories. Thus, we combine different strategies, adapting previously published studies to solve a practical case: a remote mini PC wirelessly linked with a mobile robot, and a camera sensor that implements a double triggering mechanism, one for sending commands aperiodically to a robot and another to request pose information from a camera only when required (see Figure 1). This is a first approach to tackle a more ambitious challenge in which several robotic units and several sensorial modules are managed aperiodically by the same remote centre. After presenting the theoretical framework, we report experimental results from a realistic scenario.

The main contributions of this paper are:

- Integration of event-based sensing and control techniques for nonlinear trajectory tracking of a non-holonomic robot.
- Requesting measurements based on the error estimation covariance matrix, and correcting the predicted state taking into account the PC-sensor communication delay.
- Control triggering mechanism based on state estimation, which is recalculated when a new measurement is available.
- EBSE and EBLC algorithms implemented on a PC wirelessly linked to a robot node and a camera node, experimental validation of the global strategy.

The rest of the paper is organized as follows: Section 2 presents the problem statement; Section 3 describes the event-based Lyapunov control solution; Section 4 describes the Event-Based State Estimation strategy; Section 5 details the more relevant implementation aspects; Section 6 shows experimental results; and Section 7 summarizes the contribution of the paper.

## 2. Problem Statement

The plant is a non-holonomic mobile robot locally implementing a periodic servosystem for the application of speed commands. The dynamics of the system is sufficiently rapid to be disregarded, and it is only necessary to consider the kinematic model for estimation and trajectory tracking control. The sensor node has the capacity to provide pose information using computer vision, providing at most seven measurements per second.

**Figure 2:**
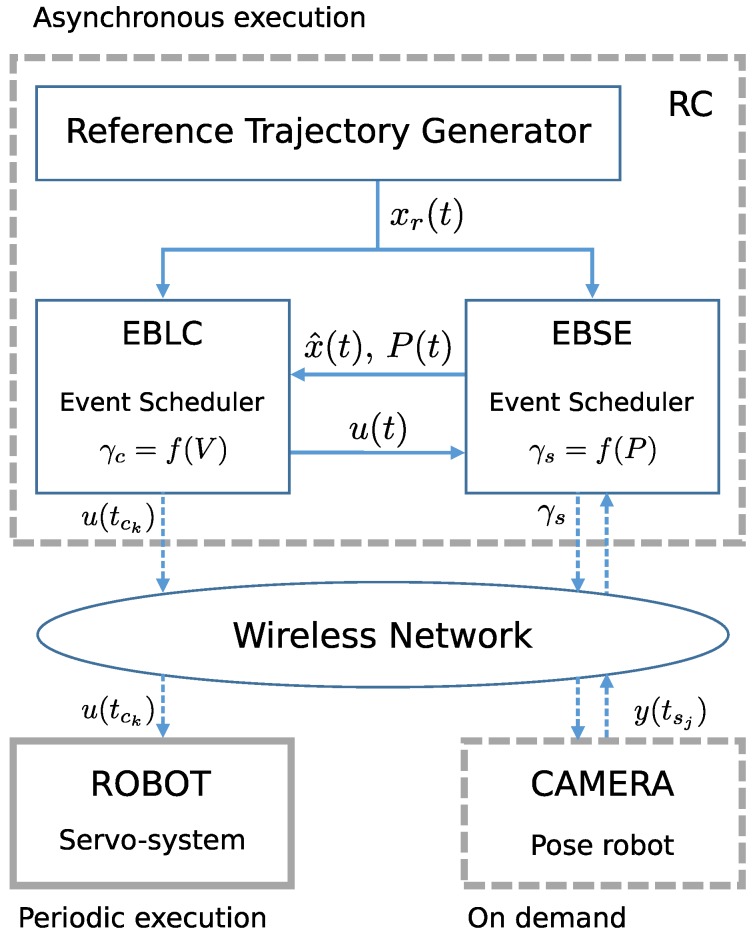
Description of the tasks carried out by the remote centre: reference trajectory generation, event-based state estimation with measurement request triggering and event-triggered control based on Lyapunov functions for asynchronous actuations on the robot.

Of the three elements shown in Figure 1, the remote centre performs the most essential and important tasks: generation of trajectory, EBLC and EBSE, as shown in Figure 2. These tasks are executed asynchronously with different event generation mechanisms.

The controller block’s missions are:

1. Implementation of aperiodic and nonlinear control law to generate linear and angular speed commands, guaranteeing system stability. For this, Lyapunov equations $V(\hat{x})$ were designed.
2. Control events generation ($t_{c_{k}}$) using current information on the estimated state vector $\hat{x}$.

The controller’s inputs are the target point $x_{r}\left(t\right)$ and the estimated pose $\hat{x}\left(t\right)$. The control law is assessed at periodic time instants with a step Δ, which is imposed by hardware limitations and the EBLC’s minimum inter-event time. The controller’s outputs are linear and angular speed commands for the robot at the triggering time instants $t_{c_{k}}$.

Variable channel delays and the plant’s response time are tackled according to the methods described in our previous study [26].

The main tasks of the estimator block are:

1. To provide a state prediction for every time step Δ. Given that the plant model is nonlinear, this task is performed using the Unscented transformation. This prediction is then corrected every time a requested measurement is received.
2. To request Measurements in order to obtain information from the sensor at the correct time instants $t_{s_{j}}$, so that the estimation error covariance matrix P(t) remains below predefined threshold conditions [24].
3. To update the prediction each time, a measurement is received, according to the acquisition times $t_{s_{j}}$ and the time elapsed until such measurements are received ($\tau_{s}$), due to image processing and communication delays.

### Robot Model

**Figure 3:**
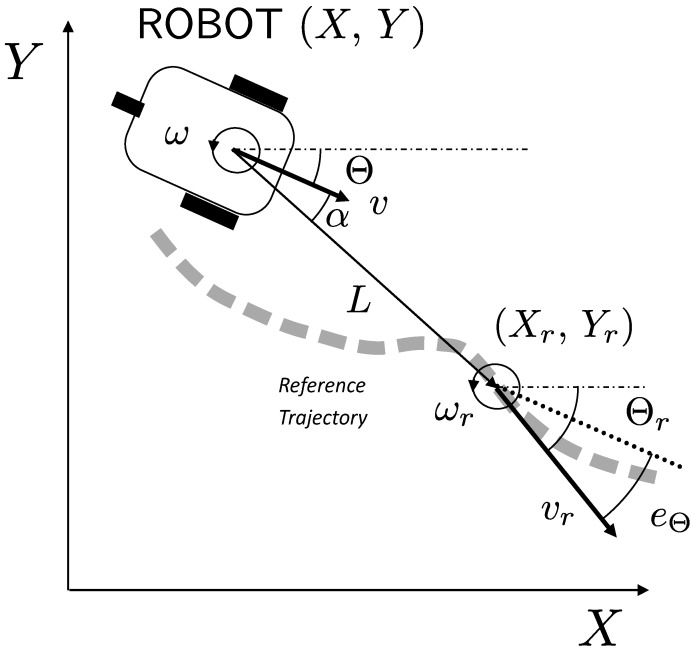
Main variables describing the trajectory tracking problem: current robot pose (X,Y,Θ), reference pose to be tracked $\left(X_{r},Y_{r},\Theta_{r}\right)$, distance error *L* and angle error α.

Figure 3 shows the main elements involved in the trajectory tracking problem, where the reference pose is characterized by $\left(X_{r},Y_{r},\Theta_{r}\right)$ coordinates and the current pose of the robot by (X,Y,Θ). The time derivative of the pose is related to the linear *v* and angular ω velocities according to the robot kinematic model, also valid for reference pose and velocities.

The robot kinematic model is described by:

(1) $$\begin{array}{cc} \dot{X}\left(t\right) & =v(t)\;\cos(\Theta (t)),\\ \dot{Y}\left(t\right) & =v(t)\;\sin(\Theta (t)),\\ \dot{\Theta }\left(t\right) & =\omega (t). \end{array}$$

All reference variables are assumed to be piecewise constant and known beforehand to the remote controller.

In addition, the following error variables should be considered: distance error *L* between the current and reference position, angle error α of the current orientation with respect to the target point, and orientation error $e_{\Theta }$ between the desired ($\Theta_{r}$) and current (Θ) orientations.

The distance error *L* and the angle error α with respect to the target point are given by:

(2) $$L(t)=\sqrt{{\left(X_{r}\left(t\right)-X\left(t\right)\right)}^{2}+{\left(Y_{r}\left(t\right)-Y\left(t\right)\right)}^{2}},$$

$$\alpha (t)=\mathrm{atan}2\left(\frac{Y_{r}\left(t\right)-Y\left(t\right)}{X_{r}\left(t\right)-X\left(t\right)}\right)-\Theta \left(t\right).\;$$

Previous studies of aperiodic control [14,27] have used Cartesian coordinates. If the vehicle is localized with a set of Cartesian variables, the target position and orientation cannot be reached asymptotically by means of smooth and time-invariant feedback control laws, due to the limitations indicated by Brockett’s result [28]. In contrast, with a polar state-space representation, a simpler approach is possible that directly allows smooth stabilization [29,30]; consequently, we decided to use polar coordinates.

Considering the kinematic model (1), the evolution of the distance and orientation errors, in polar coordinates [30], results in:

(3) $$\dot{L}\left(t\right)=-v\left(t\right)\;\cos\left(\alpha \left(t\right)\right)+v_{r}\left(t\right)\cos\left(\alpha \left(t\right)-e_{\Theta }\left(t\right)\right),\;$$

$$\dot{\alpha }\left(t\right)=-\omega \left(t\right)+v\left(t\right)\frac{\sin(\alpha (t))}{L(t)}-v_{r}\left(t\right)\frac{\sin(\alpha \left(t\right)-e_{\Theta }\left(t\right))}{L(t)},$$

$${\dot{e}}_{\Theta }\left(t\right)=\omega_{r}\left(t\right)-\omega \left(t\right).\;$$

**Assumption** **1.** All reference velocities, $v_{r}\left(t\right)$ and $\omega_{r}\left(t\right)$, are assumed to be piecewise constant and known beforehand to the remote controller.

## 3. Event-Based Control Solution

In this section, we present the control solution. We designed an aperiodic Lyapunov based controller to track the nonlinear trajectories.

### 3.1. Notation

We indicate with ‖v‖ the Euclidean norm of the vector $v\in R^{n}$. A function is said to be of class $C^{0}\left(D_{x}\right)$ if it is continuous over $D_{x}$, and it is said to be $C^{l}\left(D_{x}\right)$, l>0 if its derivatives are of class $C^{l-1}\left(D_{x}\right)$. A continuous function ρ:[0,a)→+∞,a>0 is said to be of class K if it is strictly increasing and ρ(0)=0. A Lyapunov level set is represented by $\Omega_{V_{k}}=\left\{\xi \left(t\right)\in R^{n_{x}}|V\left(\xi \left(t\right)\right)\leq V_{k}\right\}\subset D_{x}$.

### 3.2. Lyapunov Based Controller for Nonlinear Trajectory Tracking

In this section, we describe the controller used to track the nonlinear trajectory. Inspired by [30], the following linear and angular control laws are applied to (3):

(4) $$\begin{array}{cc} v(t) & =K_{v}L\left(t\right)\cos\left(\alpha \left(t\right)\right)+v_{r}\left(t\right)\cos\left(e_{\Theta }\left(t\right)\right),\; \end{array}$$

(5) $$\begin{array}{cc} \omega (t) & ={\dot{\Theta }}_{md}\left(t\right)+v_{md}\left(t\right)\left(K_{\omega }\left(K_{v}L\left(t\right)\sin\left(\alpha \left(t\right)\right)+v_{r}\left(t\right)\sin\left(e_{\Theta }\left(t\right)\right)\right)+L\left(t\right)\sin\left(\alpha \left(t\right)\right)\right), \end{array}$$

where $\theta_{md}$ is the modified desired heading angle:

(6) $$\theta_{md}\left(t\right):=\mathrm{atan}2\left(\frac{K_{v}L\left(t\right)\sin\left(\alpha \left(t\right)-e_{\Theta }\left(t\right)\right)}{v_{r}\left(t\right)+K_{v}L\left(t\right)\cos\left(\alpha \left(t\right)-e_{\Theta }\left(t\right)\right)}\right)+\Theta_{r}\left(t\right),$$

and $v_{md}$ is the modified desired linear velocity:

(7) $$v_{md}\left(t\right):=\sqrt{v_{r}{\left(t\right)}^{2}+{\left(K_{v}L\left(t\right)\right)}^{2}+2v_{r}\left(t\right)K_{v}L\left(t\right)\cos\left(\alpha \left(t\right)-e_{\Theta }\left(t\right)\right)},$$

and $K_{v}>0$ and $K_{\omega }>0$ are the tracking gains associated with the linear and angular velocities. The Lyapunov function for the resulting closed-loop system is:

(8) $$V\left(t\right)=\frac{1}{2}L{\left(t\right)}^{2}+1-\cos\left(\theta_{md}\left(t\right)-\Theta \left(t\right)\right).$$

### 3.3. Event-Based Lyapunov Control

In this section, we formulate the problem of aperiodic control applied to nonlinear systems.

Consider an autonomous nonlinear control system:

(9) $$\dot{x}\left(t\right)=f\left(x\left(t\right),u\left(t\right)\right),$$

where $x\left(t\right)\in D_{x}\subset R^{n_{x}}$ and $u\left(t\right)\in D_{u}\subset R^{n_{u}}$, both domains containing the origin.

**Assumption** **2.** *There exists a differentiable state feedback law $K:D_{x}\to D_{u}$ such that the origin of the closed-loop continuous system*

(10) $$\dot{x}\left(t\right)=f\left(x\left(t\right),K\left(x\left(t\right)\right)\right)$$

*is the unique locally asymptotically stable equilibrium point in $D_{x}$.*

From Assumption 2, converse theorems [31,32] ensure the existence of a Lyapunov function V(x(t)) for the system (10) such that:

(11) $$\begin{array}{c} \underline{\rho }\left(\|x\left(t\right)\|\right)\leq V\left(x\left(t\right)\right)\leq \bar{\rho }\left(\|x\left(t\right)\|\right),\\ \dot{V}\left(x\left(t\right)\right)=\frac{\partial V(x(t))}{\partial x}f\left(x\left(t\right),K\left(x\left(t\right)\right)\right)\leq -\rho_{1}\left(\|x\left(t\right)\|\right),\\ ∥\frac{\partial V(x(t))}{\partial x}∥\leq \rho_{2}\left(\|x\left(t\right)\|\right), \end{array}$$

with $\underline{\rho },\bar{\rho },\rho_{1},\rho_{2}\in K$.

**Assumption** **3.** *Assume that:*

*1*. The function $f\in C^{l}\left(D_{x}\times D_{u}\right)$, with l≥3.
*2*. *The functions $\underline{\rho },\rho_{1}\in K$ in (11) are such that ${\underline{\rho }}^{-1},\rho_{1}$ are Lipschitz continuous in the compact working set $\left(D_{x}\right)$.*

As described in the previous sections, we consider that the control signal is implemented in a zero-order hold (ZOH) fashion, i.e.,

(12) $$u\left(t\right)=K\left(x\left(t_{c_{k}}\right)\right),\;t\in [t_{c_{k}},t_{c_{k+1}}[,\;k\in N.$$

With this implementation, the dynamics of the sampled-data system are:

(13) $$\dot{x}\left(t\right)=f\left(x\left(t\right),K\left(x\left(t_{c_{k}}\right)\right)\right),\;t\in [t_{c_{k}},t_{c_{k+1}}[,\;k\in N.$$

We use the aperiodic proposal of [33] to present the triggering strategy. In [33], the error function g(t) is defined as:

(14) $$g\left(t\right):=f\left(x\left(t\right),K\left(x\left(t_{c_{k}}\right)\right)\right)-f\left(x\left(t\right),K\left(x\left(t\right)\right)\right),\;t\in [t_{c_{k}},t_{c_{k+1}}[,\;k\in N,$$

and the dynamics of the sample-data system are rewritten as:

(15) $$\dot{x}\left(t\right)=f\left(x\left(t\right),K\left(x\left(t\right)\right)\right)+g\left(t\right),\;t\in [t_{c_{k}},t_{c_{k+1}}[,\;k\in N.$$

**Definition** **1.** Semiglobal practical stability [34]: a system $\dot{x}\left(t\right)=f\left(x\left(t\right),K\left(x\left(t\right)\right)\right)$ is said to be semiglobally practically stable if for any (arbitrarily large) compact set $D_{x}$ and any arbitrarily small compact set $D_{V_{0}}$ including the origin, every trajectory of the system with $x\in D_{x}$ is defined for all t∈[0,∞[ and there exists T∈[0,∞[ such that $x\in D_{V_{0}}$ for all t∈[T,∞[, where $D_{V_{0}}\subset D_{x}$.

Suppose that Assumptions 2 and 3 hold for $D_{x}$ and $x\left(t_{k}\right)\in D_{x}$, where δ is a positive constant. The following triggering strategy guarantees semiglobal practical stability of the closed-loop system (13):

(16) $$t_{c_{k+1}}=\min\{t>t_{c_{k}}\;|\;\|g\left(t\right))\|>\delta \}\;.$$

Finding a lower bound of the inter-sampling time $t_{min}$ is still an open issue [33]. In order to avoid Zeno-executions, we force a $t_{min},$ taking into account the hardware constraints.

## 4. Event-Based State Estimation

The objective is to provide the controller with an accurate estimation of the state vector *x* in order to close the control loop. Since we used the unicycle kinematic model, the state vector is $x={\left[\begin{array}{ccc} X & Y & \Theta \end{array}\right]}^{T}$ and the input vector is $u={\left[\begin{array}{cc} v & \omega \end{array}\right]}^{T}$.

The control inputs change at time instants $t_{c_{k}}$ and maintain their value until the next control event $t_{c_{k+1}}$. Thus, the discrete-time system model of the robot is:

(17) $$x\left(t+\Delta \right)=x\left(t\right)+\left[\begin{array}{c} v\left(t_{c_{k}}\right)\Delta \cos\left(\Theta \left(t\right)+\omega \left(t_{c_{k}}\right)\Delta \right)\\ v\left(t_{c_{k}}\right)\Delta \sin\left(\Theta \left(t\right)+\omega \left(t_{c_{k}}\right)\Delta \right)\\ \omega (t_{c_{k}})\Delta \end{array}\right]+\eta_{x}\left(t\right),$$

where $t_{c_{k}}$ is the time of the last control event and $\eta_{x}$ is an additive Gaussian noise that takes into account possible external perturbations, with the covariance matrix:

(18) $$E\left[\eta_{x}\left(t\right)\eta_{x}^{T}\left(t\right)\right]=Q_{x}.$$

We know the speed commands $u={\left[\begin{array}{cc} v & \omega \end{array}\right]}^{T}$ generated by the controller. However, Equation (17) defines an ideal kinematic model that does not account for the real robot dynamics, so the actual linear and angular speeds $u_{d}={\left[\begin{array}{cc} v_{d} & \omega_{d} \end{array}\right]}^{T}$ are not precisely known. In order to account for speed error, we consider the noise vector $\eta_{u}$, defined as:

(19) $$\eta_{u}\left(t\right)=u_{d}\left(t\right)-u\left(t_{c_{k}}\right)$$

with the covariance matrix:

(20) $$E\left[\eta_{u}\eta_{u}^{T}\right]\approx Q_{u}.$$

Since model (17) is nonlinear, the prediction stage of the filter is carried out periodically by an Unscented Kalman Filter (UKF). With this algorithm, we obtain an estimated state vector and an approximation of the estimation error covariance matrix *P*:

(21) $$P\approx E\left[\left(x-\hat{x}\right){\left(x-\hat{x}\right)}^{T}\right].$$

Let *h* be the function that computes the estimation prediction using the Unscented Transformation:

(22) $$\left({\hat{x}}^{-}\left(t+\Delta \right),\;P^{-}\left(t+\Delta \right)\right)=h\left({\hat{x}}^{-}\left(t\right),\;u\left(t\right),\;P^{-}\left(t\right),\;Q_{x},Q_{u}\right).$$

The EBSE schedules measurement events at given time instants $t_{s_{j}}$ at external sensors. Measurement requests are denoted with the symbol $\gamma_{s}$, so $\gamma_{s}=1$ means the remote centre is requesting a measurement, and $\gamma_{s}=0$ otherwise.

The sensor must then detect the robot pose at these time instants, but the measurements contain noise. The output equation of the system is

(23) $$y\left(t_{s_{j}}\right)=Cx\left(t_{s_{j}}\right)+\eta_{y}\left(t_{s_{j}}\right),$$

where C=I is the output matrix and $\eta_{y}$ is a discrete uncorrelated Gaussian noise with the covariance matrix:

(24) $$E\left[\eta_{y}\left(t_{s_{j}}\right){\eta_{y}}^{T}\left(t_{s_{j}}\right)\right]=R\left(t_{s_{j}}\right)\;.$$

The correction stage of the filter is performed for every time instant $t_{s_{j}}$ that a measurement is taken:

(25) $${\hat{x}}^{+}\left(t_{s_{j}}\right)={\hat{x}}^{-}\left(t_{s_{j}}\right)-P^{-}\left(t_{s_{j}}\right)C^{T}{\left(CP^{-}\left(t_{s_{j}}\right)C^{T}+R\left(t_{s_{j}}\right)\right)}^{-1}\left(y\left(t_{s_{j}}\right)-C{\hat{x}}^{-}\left(t_{s_{j}}\right)\right),$$

and the a posteriori covariance matrix is:

(26) $$P^{+}\left(t_{s_{j}}\right)=P^{-}\left(t_{s_{j}}\right)-P^{-}\left(t_{s_{j}}\right)C^{T}{\left(CP^{-}\left(t_{s_{j}}\right)C^{T}+R\left(t_{s_{j}}\right)\right)}^{-1}CP^{-}\left(t_{s_{j}}\right).$$

### 4.1. Sensor Event Triggering

A measurement is requested when the estimation uncertainty $P^{-}$ is sufficiently large. The triggering criteria used in this study was first proposed in [24] and has been tested in a mobile robot in [35].

There are two different triggering conditions: the first is based on the estimation distance error and is an adaptive condition. Considering that the estimated distance $\hat{L}$ to the target point $\left(X_{r},\;Y_{r}\right)$ is defined as:

(27) $$\hat{L}=\sqrt{{\left(X_{r}-\hat{X}\right)}^{2}+{\left(Y_{r}-\hat{Y}\right)}^{2}}.$$

The distance condition is:

(28) $$P_{1,1}+P_{2,2}>D_{\mathrm{trk}}^{2}+K_{D}^{2}{\hat{L}}^{2},$$

where $P_{i,i}$ are the diagonal elements of *P*, $D_{\mathrm{trk}}>0$ is the desired distance error threshold during tracking time and $K_{D}>0$ is a constant that is used to allow more uncertainty when the target point is located far from the current robot position. It is wise to allow this uncertainty for high values of *L* since the controller will guide the robot towards its target, and the control actions will be fairly similar around a certain area, so knowing the precise robot location is not really necessary.

The second condition is based on orientation uncertainty. This should not be greater than ${\tilde{\Theta }}_{\mathrm{thr}}$, so the orientation condition is written as:

(29) $$P_{3,3}>{\tilde{\Theta }}_{\mathrm{thr}}^{2}.$$

Whenever either of these two conditions becomes true, a correction is required and so a measurement must be requested. The combination of these two conditions can be written as a single statement:

(30) $$\left(P_{1,1}+P_{2,2}>D_{\mathrm{trk}}^{2}+K_{D}^{2}{\hat{L}}^{2}\right)\vee \left(P_{3,3}>{\tilde{\Theta }}_{\mathrm{thr}}^{2}\right).$$

Further discussion about these parameter adjustments can be found in [35].

### 4.2. Control Design Dependent on State Estimation

The problem of output feedback stabilization with a state observer can be solved with a linear observer and a linear control law. If the linear plant is detectable and stabilizable, the separation principle allows for designing the controller gain and observer gain independently. However, this cannot be directly applied to nonlinear systems. In [36], a separation principle is proved for a particular class of nonlinear systems. An output feedback controller using a fast high-gain observer makes it possible to achieve the desired performance by means a state feedback controller. In [37], the authors focus on nonlinear systems whose linear approximation is not stabilizable. They designed a solution that locally asymptotically stabilizes the single-input nonlinear affine system by a state estimate feedback law given by considering a Kalman observer associated with the bilinear system. In [38], a separation principle is analysed for time-varying dynamical systems, where the matrices *A*, *B*, *C*, *D* of the linear state space model are time dependent. Thus, the observer and controller are designed in the continuous time domain.

In general, the separation principle does not hold for event-triggered control systems [39]. In [40], the stabilization of a nonlinear control system using dynamic output feedback is analysed theoretically. Two triggering mechanisms are proposed, one to control actions and the other to update the estimated state by asking the sensor unit for measurements, but guaranteeing global asymptotic stability of the closed-loop. Maintaining a sufficiently small error between the current output value and the last output sample is the condition to decouple the two control and sensing triggering mechanisms. A Lyapunov function with two terms is proposed, one related to the estimation error and the other to the plant state, but their derivatives are bounded independently. In addition, a send-on delta solution is adopted for the “output sampling rule” (sensor) and a similar one for the input sampling rule (controller).

In our nonlinear system case, the Lyapunov function designed for stability purposes is based on the predicted state estimation vector, which is re-evaluated when a new measurement is available. Consequently, (14) is replaced by:

(31) $$\hat{g}\left(t\right):=f\left(\hat{x}\left(t\right),K\left(\hat{x}\left(t_{c_{k}}\right)\right)\right)-f\left(x\left(t\right),K\left(x\left(t\right)\right)\right),\;t\in [t_{c_{k}},t_{c_{k+1}}[,\;k\in N.$$

The estimation error is bounded between the different measurements thanks to the EBSE implemented. Thus, our EBLC neglects the estimation error between measurements. The main drawback of this strategy is that bounded peaks may be obtained in the Lyapunov function when the estimation error appears. The control triggering instants are calculated by:

(32) $$t_{c_{k+1}}=\min\{t>t_{c_{k}}\;|\;\|\hat{g}\left(t\right))\|>\delta \}\;.$$

On the other hand, we achieve longer times between control updates. The main point is that our strategy does not change the stability properties of the aperiodic system; it only changes the accuracy of the model used by our EBLC [33].

## 5. Implementation

Our objective was to compute the control commands *u* using $\hat{x}$, which has an estimation error covariance matrix *P* that should be bounded by the triggering condition (30).

In a practical implementation, we must consider the time delays in our system. Let $\tau_{n}$ be the network delay, i.e., the time it takes to transmit a message through the communication network. From the sensor, we must consider the time $\tau_{s}$ taken between a measurement acquisition and the instant it reaches the remote centre. This time is required for image acquisition and processing, and network communication. For the robot, we must also consider the time $\tau_{c}$ taken between a command being sent and its observable effect on the robot. In this case, $\tau_{c}$ is the sum of the network delay and the response time of the robotic platform.

In order to determine the length of these delays, we require clock synchronization between the sensors, robot and remote centre. Considering our time step Δ=10ms, synchronization within this order of magnitude can be achieved using standard time synchronisation protocols such as Precision Time Protocol (PTP) or even Network Time Protocol (NTP).

**Figure 4:**
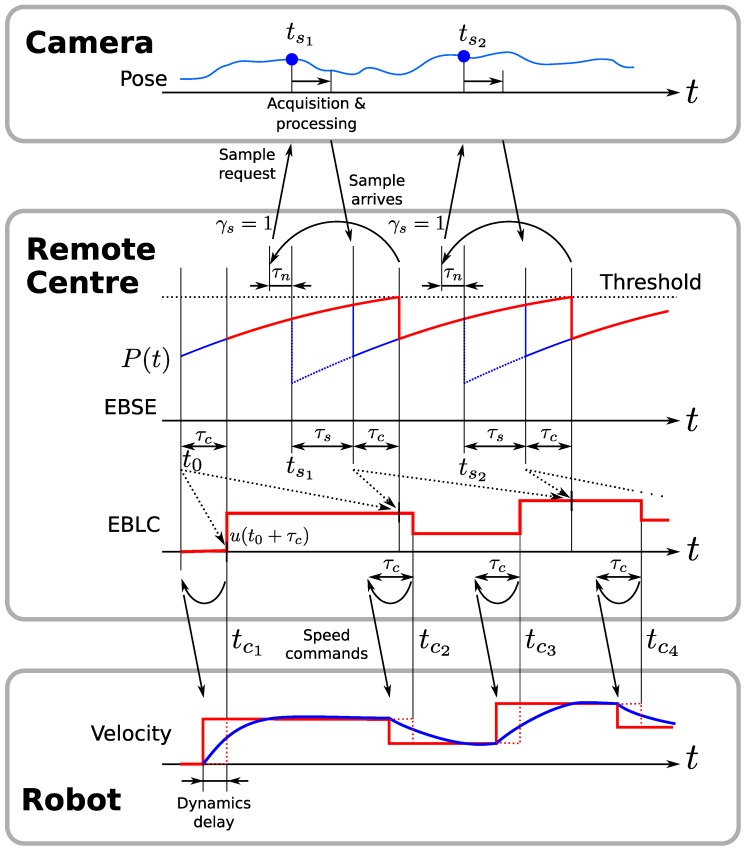
Diagram showing communications between the remote centre, sensor and robot, explaining the strategy to compensate for delays.

Given the control delay $\tau_{c}$, it is desirable to know *u* at least $\tau_{c}$ seconds ahead in order to send it to the robot in advance and compensate for the network delay and motor dynamics. At each time *t*, the remote centre transmits $u(t+\tau_{c})$ if and only if $\gamma_{c}\left(t+\tau_{c}\right)=1$. Consequently, the estimation should have a bounded uncertainty for all time instants at which the control is computed (see Figure 4).

At a point $t_{s_{j}}+M\Delta$, the uncertainty threshold (30) will be reached, and no further control commands should be computed unless a new measurement is received. This measurement must be received at least $\tau_{c}$ seconds before $t_{s_{j}}+M\Delta$, so that the following control commands can be computed with an estimation uncertainty below the predefined threshold. Furthermore, in order to obtain this measurement early enough, it must be requested taking into account the acquisition and network delays that will occur after the request is sent. Consequently, the measurement must be requested with a total of $\tau_{n}+\tau_{s}+\tau_{c}$ time in advance.

**Figure 5:**
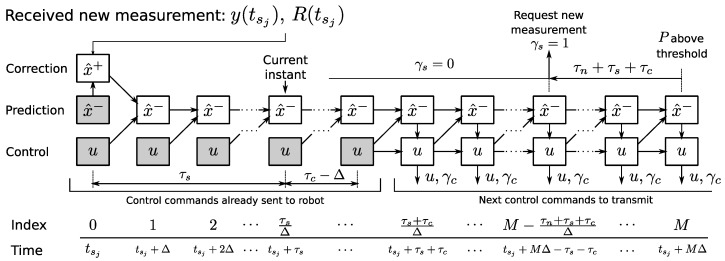
Table containing the computed estimation and control values.

Once the measurement taken at time $t_{s_{j}}$ arrives at time $t_{s_{j}}+\tau_{s}$, it is possible to predict the state and compute the control commands for all times until the next threshold crossing instant. To do so, it is necessary to store the values of ${\hat{x}}^{-}\left(t_{s_{j}}\right)$ and $P^{-}\left(t_{s_{j}}\right)$. Then, Equations (25) and (26) are applied to them and the a posteriori estimation is propagated to the present time instant using Equation (22) and the stored output commands *u* up to time $t_{s_{j}}+\tau_{s}+\tau_{c}$, which have been already sent to the robot. Subsequent outputs are calculated using the respective state prediction, ${\hat{x}}^{-}\left(t\right)$. Figure 5 illustrates the implementation of this algorithm using a table where the outputs and state predictions are stored and computed iteratively.

This table is constructed every time a measurement arrives, and it stores all values of $\hat{x}$ and *u* in case $\tau_{s}$ is different from what is expected. The grey cells in Figure 5 represent pre-calculated control commands that have already been sent to the robot, as well as the corresponding a priori estimation at the appropriate sampling time $t_{s_{j}}$. In contrast, all white cells must be computed anew.

## 6. Experimental Tests

### 6.1. Set-Up

The tests were performed using a Pioneer P3-DX robot (Omron Adept, Amherst, NH, USA [41]) with additional electronics such as it is described in [42]. A mini PC (model NUC5i3RYH [43]) with a Core i3 processor (Intel Corp., Santa Clara, CA, USA) and 4 GB of RAM implemented the remote centre. The sensor node included an identical mini PC attached to a Kinect RGB camera (version 2, Microsoft, Redmond, WA, USA. Product specs can be found at [44]), which has a resolution of 1920×1080. Both PCs were running Ubuntu (version 12.04, Canonical Ltd., London, UK) as their operating system, and clock synchronisation between them was achieved using the standard NTP tools (version 4.2.6) provided by the operating system.

**Figure 6:**
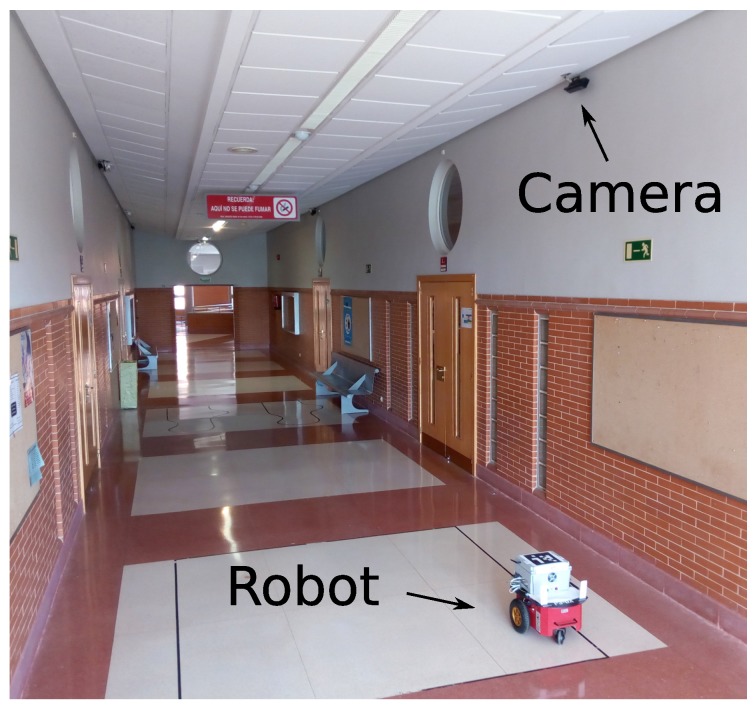
Picture of the working area with a P3-DX robot and Kinect 2 camera sensor.

The camera was placed at a height of 3 m as can be seen in Figure 6.

A marker was placed on top of the robot and its pose was detected using the AprilTags fiducial system (version 2, University of Michigan, MI, USA) [45]. This software provides the pixel coordinates of the marker corners found in an image. A pose is then obtained by projecting these coordinates onto the ground plane using the geometric pin-hole camera model. The position is the mean point of the projected corners, and the orientation is computed using the arctangent function.

The marker side length is 173mm, and thus the diagonal length is 245mm. Depending on the robot’s position, this marker is seen by the camera with a minimum diagonal size of 58 pixels, or a maximum of 87 pixels. Although the camera has the capacity to obtain up to 30 frames per second, processing such frames in order to detect the marker pose is a slow computation process for the mini PC. Since the image processing times were long and variable, we reduced the search area of the image to the surroundings of the last detection, but even with this method, the maximum rate achieved was seven measurements per second.

Considering the image quality and the repeatability of measurements, we assumed a detection error of two pixels for each marker’s corner coordinate. Then, the associated measurement noise matrix *R* was obtained by propagating this pixel uncertainty to the measurement computation using the Unscented Transformation.

Process noise and input noise covariance matrices were determined empirically:

(33) $$Q_{x}=\left[\begin{array}{ccc} 10^{-8} & 0 & 0\\ 0 & 10^{-8} & 0\\ 0 & 0 & 10^{-8} \end{array}\right];\;Q_{u}=\left[\begin{array}{cc} 0.2 & 0\\ 0 & 0.4 \end{array}\right].$$

Estimator threshold parameters were set to $D_{\mathrm{trk}}=0.25\;m$, $K_{D}=1/8$ and ${\tilde{\Theta }}_{\mathrm{thr}}=5^{\circ }$. The controller threshold parameter was δ=0.1

The real robot position was approximated by allowing the camera node to obtain and store measurements continuously, applying an Unscented Kalman Smoother [46] after the tests. The state estimation $x_{p}$ obtained in this way uses all available measurements (past and future), and was used here to approximate the estimation (*D*) and tracking (*L*) error:

(34) $$\begin{array}{cc} D & =\sqrt{{\left(\hat{X}-X_{p}\right)}^{2}+{\left(\hat{Y}-Y_{p}\right)}^{2}}, \end{array}$$

(35) $$\begin{array}{cc} L & =\sqrt{{\left(X_{r}-X_{p}\right)}^{2}+{\left(Y_{r}-Y_{p}\right)}^{2}}. \end{array}$$

Concerning delay times, the network delay for a single message was typically lower than the discretisation period Δ, so we considered $\tau_{n}=\Delta =10\;\mathrm{ms}$. The observed measurement delay was $\tau_{s}\approx 145\;\mathrm{ms}$, with a standard deviation of 10ms. Finally, we considered a delay for the robot dynamics of $\tau_{c}=200\;\mathrm{ms}$.

### 6.2. Results

In order to compare the performance obtained with the event-based method explained in Section 3 and Section 4, four different sets of tests were performed. For each experiment, the reference trajectory was a figure eight shape followed by a straight line, which features several speed changes. The event-based implementation was executed using the parameters described in Section 6.1. Additionally, several periodic implementations were tested.

In the first one, the control inputs were computed at every discretisation step, so that it emulates a continuous-time controller. Sensor events were requested continuously, so that the remote centre received approximately seven measurements per second. The second implementation is similar to the one described above, but control and sensor updates were scheduled periodically with the same time period. Therefore, the time period was set to T=150ms. Finally, in order to get approximately the same amount of updates than in the event-based implementation, another set of experiments was performed using T=700ms.

For each of these implementations, five runs were executed, and the results are summarized in Table 1. The number of control and estimation updates is shown as well as the estimation (*D*) and tracking (*L*) Root Mean Squared errors (RMS). The mean result and the standard deviation are shown for each case.

In the first two rows (fastest and T=150ms implementations), we see that the results are almost identical. They achieve the best performance, but using a high number of updates. However, with the aperiodic implementation, we obtained a similar tracking error but with a reduced number of updates (less than one fourth).

If, instead, we wanted to reduce the number of updates by increasing the period (T=700ms), we see that the tracking performance becomes much worse. Additionally, these tests were the most unpredictable so they had the highest standard deviation.

Concerning estimation, the aperiodic implementations showed a slightly higher *D* than the T=700ms case. The reason is that the adaptive triggering explained in Section 4 sometimes allows having more uncertainty and fewer measurements as long as it does not cause the tracking performance to deteriorate.

**Figure 7:**
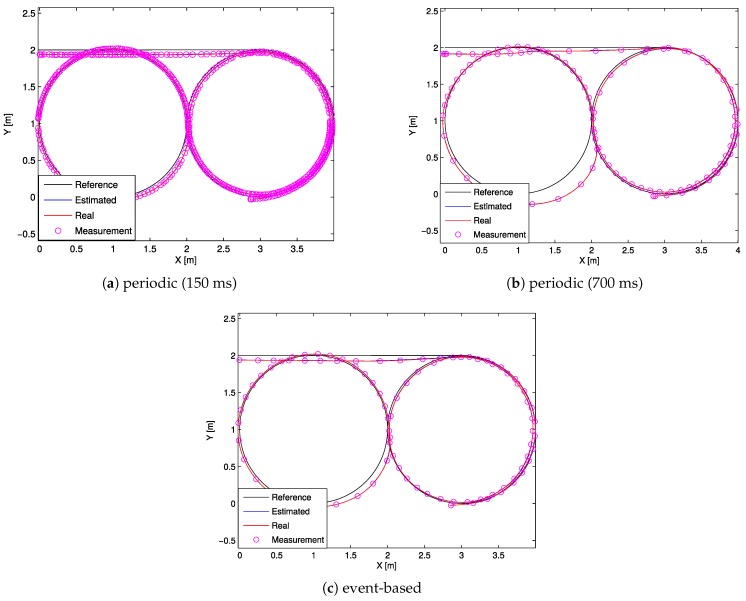
Robot trajectory developed during the tests.

In order to further explain these results, the remaining part of this section presents three representative instances of the performed experiments. Figure 7 shows the trajectory performed in the tests using periodic (with periods T=150ms and T=700ms) and event-based implementation. Although the number of measurements was significantly different, the tracking trajectory in the event-based implementation remained very similar to the 150ms periodic case. However, using the same number of updates, in the 700ms periodic case (see Figure 7b), the trajectory gets severely distorted, since the controller does not react immediately to changes in the reference.

**Figure 8:**
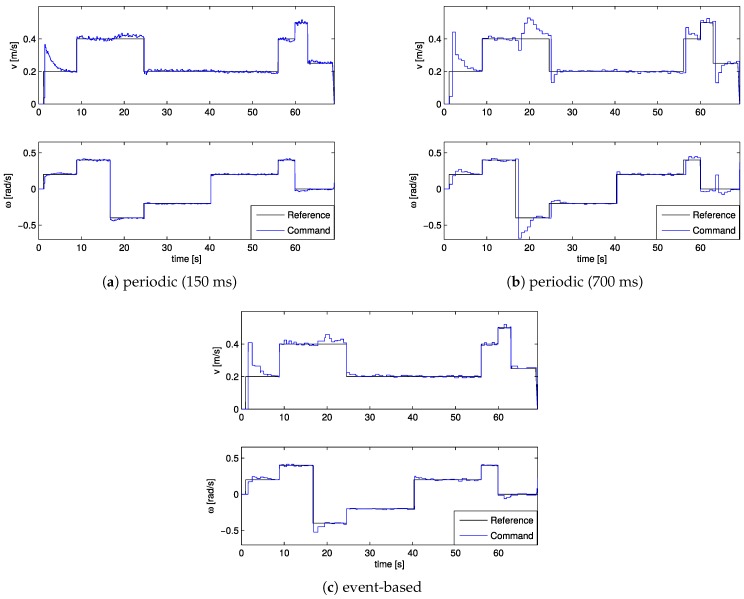
Reference and speed commands sent to the robot.

This is better shown in Figure 8, which gives the different speed profiles applied to the robot, as well as the ideal speed of the reference trajectory. Figure 8a shows smoother curves, while, in Figure 8b,c, the curve is edgy. However, the event-based implementation is more damped and has a shorter setting time on every reference change because the actuations are triggered at more appropriate instants, in contrast to this periodic case.

**Figure 9:**
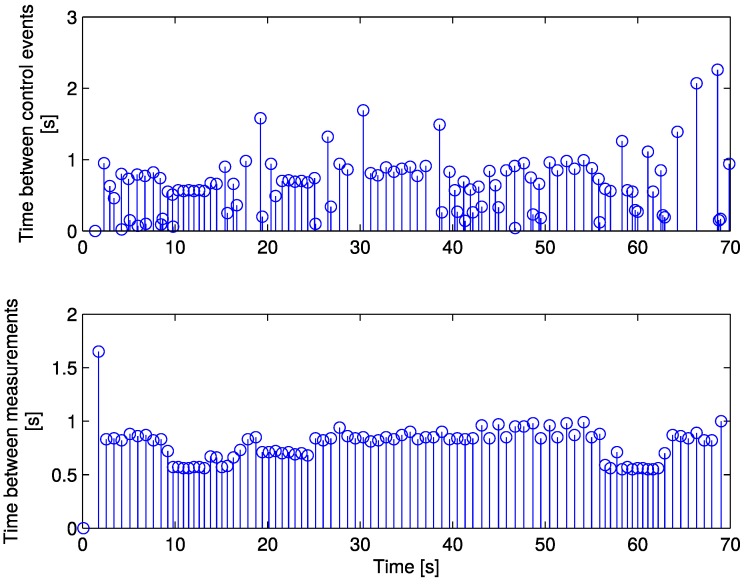
Time between controller and sensor events.

Both sensor and control event times related to the aperiodic case of Figure 7c and Figure 8c are illustrated in Figure 9. Each stem represents an event, where its horizontal position corresponds to the time instant when it occurred, and its height represents the time difference with the previous event.

Sensor events are triggered according to the uncertainty increase rate, which is tied to robot speed, but since the controller and estimator have different triggering criteria, the control events do not necessarily coincide with the sensor ones.

The minimum time between consecutive control events is $t_{c_{k}+1}-t_{c_{k}}=\Delta =10\;\mathrm{ms}$, which justifies the use of this time step for discretisation.

**Figure 10:**
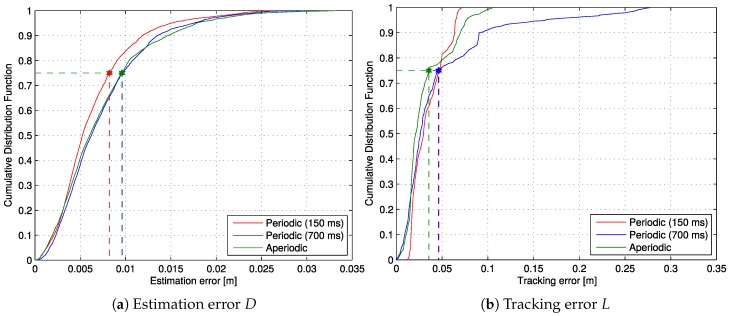
Estimation and tracking error cumulative distribution functions.

Figure 10a shows the Cumulative Distribution Function (CDF) of the estimation error, as well as the third quartile error (75 percentile). As expected, the periodic test with T=150ms yielded a smaller estimation error than the other two cases since it is obtained using much more information from the sensor. However, if we compare the tracking error (*L*) in Figure 10b, the result obtained with the event-based implementation has the lowest third quartile error, and its curve is pretty close to the 150ms case, indicating that the improvement in estimation precision does not necessarily result in improved guidance.

Figure 10a shows the Cumulative Distribution Function (CDF) of the estimation error, as well as the third quartile error (75 percentile). As expected, the periodic test with T=150ms yielded a smaller estimation error than the other two cases since it is obtained using much more information from the sensor. However, if we compare the tracking error (*L*) in Figure 10b, the result obtained with the event-based implementation has the lowest third quartile error and its curve is pretty close to the 150ms case, indicating that the improvement in estimation precision does not necessarily result in improved guidance.

## 7. Conclusions

This paper describes the theoretical foundations and implementation details for a mechanism to remotely and asynchronously control a robot tracking a nonlinear trajectory, using a camera as a localization sensor.

A mini PC serves as a remote centre and contains both the event-based state estimation, which triggers measurement requests by evaluating the estimation error covariance matrix, and the event-based Lyapunov controller.

The control law is calculated from the estimated robot kinematics, knowing that the covariance matrix of the estimation error is bounded by given thresholds. Every time a new pose measurement is received, with its corresponding time stamp, the predicted estimation is updated and the control is re-evaluated.

Our solution takes into account the abovementioned theoretical aspects as well as practical issues such as communication network delays, actuation time delays caused by robot dynamics, and image acquisition and processing times in order to obtain pose measurements.

Although the results presented here were obtained for a particular NCS application, with specific parameter values for EBSE and EBLC, the conclusion is clear: the combination of event-based systems, both to request measurements and update the actuation on the robot speed, significantly reduce sensor and communications activity, while maintaining an acceptable estimation error and yielding a tracking performance comparable to periodic implementation.

To validate the theoretical proposal, a considerable set of experimental tests with aperiodic and different periodic alternatives were carried out. The conclusion of our event-based sensing and control solution for the described remote robot application is that using our aperiodic proposal, in respect of the periodic solution, taking as reference the highest sampling rate of the sensor, we obtain a mean tracking error increase of 8.3% but with only 22% of the total number of updates. However, if we relax the sampling time to achieve a number of updates close to the event-based case, the mean tracking error degrades 59.5%. This is why we highlight the interest of combining event-based control with event-based state estimation as a proof of concept for event-driven control systems beyond the specific example of remote robot guidance.

## Figures and Tables

**Table 1:** Comparison of estimation and control performance between the time-driven and event-driven implementations. Four cases of study and five runs per case.

	# Control Updates		# Sensor Updates		*D* (mm rms)		*L* (mm rms)	
	Mean	St. dev.	Mean	St. dev.	Mean	St. dev.	Mean	St. dev.
Periodic (fastest)	6428	–	454	–	7.77	0.46	37.05	1.78
Periodic (150ms)	429	–	429	–	7.69	0.22	38.89	1.09
Periodic (700ms)	92	–	92	–	9.17	0.55	63.97	14.57
Event based	94.17	8.97	83	5.8	10.21	0.98	40.11	7.68

## Supplementary Materials

A video showing the results of one of the performed experimental tests is available online at http://www.geintra-uah.org/demos#event17.

## Abbreviations

The following abbreviations are used in this manuscript:

CDF	Cumulative distribution function
EBLC	Event-based Lyapunov controller
EBSE	Event-based state estimator
NCS	Networked control system
NTP	Network time protocol
PTP	Precision time protocol
RMS	Root mean squared error
UKF	Unscented Kalman filter
WSN	Wireless sensor network
